# Management of volumetric muscle loss in a dog using bioactive glass–alginate–cellulose composite and a transpositional flap: a case report

**DOI:** 10.1007/s11259-026-11557-x

**Published:** 2026-10-09

**Authors:** Alina Zăvoi, Tom Ablassmaier, Bianca Pop, Ștefana Mureșan, Andrada Negoescu, Felix Lucaci, Robert Purdoiu, Alexandra Feraru, Klara Magyari, Lucia Bel, Mădălina Dragomir, Liviu Oana, Marian Taulescu, Alexandra Dreancă

**Affiliations:** 1 Faculty of Veterinary Medicine, University of Agricultural Science and Veterinary Medicine, Cluj-Napoca, 400372 Romania; 2 Nanostructured Materials and Bio-Nano-Interfaces Center, Interdisciplinary Research Institute on Bio-Nano-Sciences, Babeș-Bolyai University, Cluj-Napoca, 400271 Romania; 3 Doctoral School in Physics, Faculty of Physics, Babeș-Bolyai University, M. Kogălniceanu 1, Cluj-Napoca, 400084 Romania; 4 INSPIRE Research Platform InfoBioNano4Health & Biomedical Imaging, Babeș-Bolyai University, Cluj-Napoca, 400084 Romania

**Keywords:** Bioactive glass, Alginate, Cellulose, Volumetric muscle loss

## Abstract

This case report describes the management of a complex volumetric muscle loss (VML) injury in a 3-year-old mixed-breed dog following severe trauma to the right forelimb, which resulted in extensive multi-muscle defects that were unsuitable for primary closure. After initial stabilization and surgical debridement, reconstruction was achieved using a transpositional flap, while a gold nanoparticles–bioactive glass–alginate–cellulose (BGAuSP-Alg-MCC) composite was implanted to fill the dead space and support tissue regeneration. Postoperative healing was evaluated through clinical follow-up, computed tomography performed immediately and at 1 and 3 months postoperatively, as well as histological and immunohistochemical analyses. Imaging demonstrated progressive integration of the biomaterial, restoration of tissue density comparable to adjacent muscle, and absence of major complications. Histological evaluation demonstrated moderate inflammation and granulation tissue formation, containing numerous blood capillaries (neoangiogenesis), confirmed by CD31 and α-Smooth muscle actin (SMA) immunolabelling. Functional recovery was favorable, with preserved limb mobility and normal gait. These findings provide preliminary evidence of local biocompatibility and support the feasibility of incorporating the BGAuSP-Alg-MCC composite into a multimodal surgical management approach for extensive volumetric muscle loss in canine patients.

**Graphical Abstract:**

**Figure:**
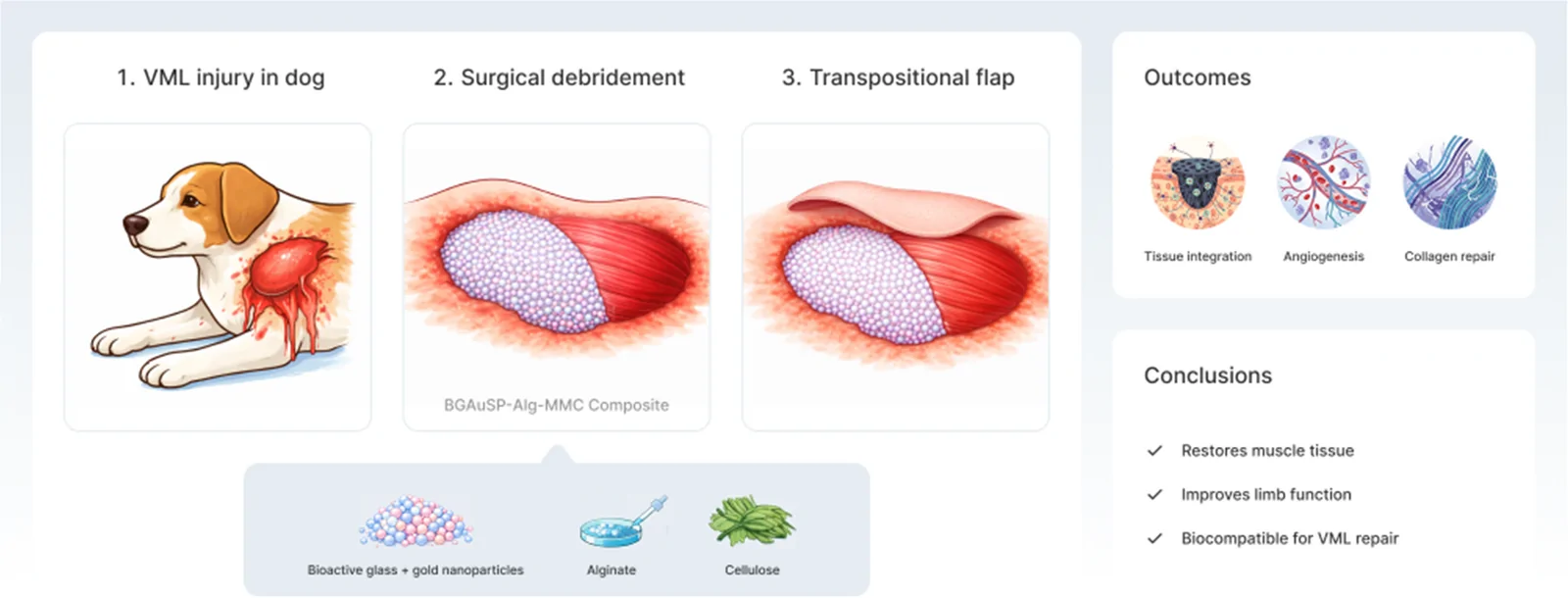

**Supplementary Information:**

The online version contains supplementary material available at https://doi.org/10.1007/s11259-026-11557-x.

## Background

Skeletal muscle regeneration after injury is a complex and dynamic process driven primarily by satellite cells (Järvinen et al. 2007; Collins et al. 2007; Lin et al. 2007). In severe trauma, the intrinsic healing capacity can be overwhelmed, resulting in volumetric muscle loss (VML), fibrosis, and functional impairment. Traumatic extremity injuries and limb-salvage procedures can cause VML, and although surgical techniques such as muscle flaps or tissue transposition are commonly employed, few clinical studies specifically address treatment outcomes. Regenerative medicine offers promising strategies to enhance repair and restore muscle structure and function in these challenging cases (Perez-Terzic and Childers 2014).

Several therapeutic strategies have been investigated for the treatment of VML in vivo, including autologous tissue grafting, surgical reimplantation, biomaterial-based approaches incorporating cells or growth factors, and stem cell transplantation (Shi et al. 2009; Corona et al. 2013, 2017; Oshima et al. 2014; Goldman et al. 2018; Qiu et al. 2018). Many of these approaches rely on the use of cellular or molecular factors to enhance tissue regeneration. In contrast, the regenerative potential of biomaterials used without additional cells or growth factors appears to be more limited. For example, devitalized skeletal muscle extracellular matrix (ECM) scaffolds have shown insufficient restoration of large muscle defects when used alone, partly because they do not adequately promote the recruitment and activation of endogenous satellite cells (Garg et al. 2014; Aurora et al. 2015).

Biomaterials with tailored composition, structure, and mechanical properties are widely investigated for soft-tissue regeneration because they can influence cell adhesion, differentiation, and tissue remodeling. An ideal soft-tissue scaffold should be biocompatible, support cell adhesion, elicit minimal inflammation and toxicity, undergo appropriate biodegradation, and provide suitable porosity (Sindhi et al. 2025).

Natural carbohydrate-based polymers, including cellulose, chitosan, alginate, and hyaluronic acid, are widely used in biomedical applications because of their biocompatibility and biodegradability (Serafin et al. 2023; Sindhi et al. 2025). Cellulose is a linear polysaccharide present in plants and several other organisms and is used in multifunctional scaffolds because of its mechanical properties, biocompatibility, low toxicity, and relatively low cost (Seddiqi et al. 2021). Alginate, derived from brown seaweed, forms ionically cross-linked hydrogels that maintain a moist environment and are used for cell encapsulation, tissue repair, and drug delivery (Ahmad Raus et al. 2021). Bioactive glasses, initially developed for bone regeneration, are also being investigated for soft-tissue repair because of their potential to support angiogenic and regenerative responses (Zhu et al. 2025; Zăvoi et al. 2025). Nevertheless, scaffold-mediated remodeling may differ across anatomical sites, particularly in injuries involving combinations of bone, muscle, and tendon. Extensive post-traumatic skin defects in companion animals remain challenging and may require serial debridement, infection control, protection of deeper structures, and reconstructive techniques such as grafts or flaps (Williams 2009).

In extensive traumatic injuries, donor-site availability may be limited (Beier et al. 2006; Mase et al. 2010), and some limb-salvage failures ultimately require amputation and prosthetic rehabilitation (Turner and Badylak 2012). These limitations support continued investigation of off-the-shelf biomimetic materials intended to assist skeletal-muscle reconstruction in large defects (Mase et al. 2010).

The gold nanoparticles–bioactive glass–alginate–cellulose (BGAuSP-Alg-MCC) composite was selected on the basis of previous experimental characterization and reported findings of cellular infiltration, angiogenesis, and myotube formation, with no evidence of cytotoxicity, biomaterial-induced tissue necrosis, or neutrophilic inflammation, supporting the biocompatibility of the material in muscle tissue (Feraru et al. 2025). In the present clinical case, the composite was used as an adjunct to reconstructive surgery in a complex defect involving the deltoid, lateral triceps brachii, and infraspinatus muscles. To our knowledge, this is the first reported canine case combining this composite with a transpositional flap for an extensive multi-muscle defect unsuitable for primary closure. The aim was to describe the surgical approach and short-term clinical, computed tomography, histological, and immunohistochemical findings; the single-case design was not intended to establish biomaterial-specific efficacy.

## Case presentation

### History and clinical findings

A 3-year-old, previously healthy, spayed mixed-breed dog (30 kg) was presented to the emergency service at the University of Agricultural Sciences and Veterinary Medicine of Cluj-Napoca approximately 1 h after being severely injured by a weed trimmer. The animal was transported to the hospital by a Non-Governmental Organization (NGO), as the owner was unable to afford the required emergency care.

Upon presentation to the emergency service, the dog was in lateral recumbency and minimally responsive. The mucous membranes were pale, and the capillary refill time was undetectable. The heart rate was 200 beats/min with a regular rhythm, the respiratory rate was 80 breaths/min, and the body temperature was 36.6 °C. Severe trauma of the right upper forelimb was identified, characterized by multiple puncture wounds, deep lacerations, and active hemorrhage.

Initial resuscitation included fluid therapy with isotonic crystalloids (30 mL/kg IV; 0.9% NaCl) and active warming with a forced-air device until body temperature reached 37.5 °C. Empirical antimicrobial therapy with amikacin (15 mg/kg IV q24h) was initiated before culture results were available. Amikacin monotherapy does not provide comprehensive streptococcal or anaerobic coverage; therefore, the initial antibiotic treatment in this case should not be interpreted as broad-spectrum coverage. Analgesia was provided with a fentanyl bolus (3 µg/kg IV; Fentadon 50 µg/mL, Dechra, Netherlands) followed by a constant-rate infusion (5 µg/kg/h IV). The initial hematologic and biochemical workup included determination of PCV, total plasma protein, and activated clotting time, along with a venous biochemistry profile encompassing blood gas values, major electrolytes (sodium, potassium, chloride, ionized calcium, ionized magnesium), glucose, lactate, BUN, and creatinine concentrations. Hematological analysis revealed a mild leukocytosis with neutrophilia and an elevated plasma lactate concentration of 3 mmol/L (27 mg/dL; reference interval < 2.5 mmol/L [< 22.5 mg/dL]), while all other hematological parameters were reported within normal limits.

### Surgical procedure

The study was approved by the Ethics Committee of the University of Agricultural Sciences and Veterinary Medicine, Cluj-Napoca, Romania (approval no. 423/22.01.2024) and conducted in accordance with local regulations. Written informed consent was obtained from the owner and the NGO. The dog remained hospitalized throughout recovery due to its remote home location and the NGO’s lack of suitable temporary housing.

After 24 h of stabilization and intensive monitoring, the dog underwent surgical intervention under general anesthesia. Cefotaxime (25 mg/kg IV q12h; Cefotax 1 g, The Egyptian International Pharmaceutical Industries Company, Egypt) was administered 30 min before surgery as perioperative antimicrobial coverage and was subsequently continued in combination with the ongoing amikacin therapy (15 mg/kg IV q24h). Meloxicam (0.2 mg/kg IV; Metacam, Boehringer Ingelheim) was administered for perioperative analgesia. Methadone (0.2 mg/kg IM; Insistor 10 mg/mL, Richter Pharma, Wels, Austria) was administered as premedication, followed 20 min later by dexmedetomidine (0.02 mg/kg IV; Dexdomitor 0.5 mg/mL, Orion Pharma) and ketamine (2 mg/kg IV; Ketomidor 10%, Richter Pharma, Austria). Anesthetic induction was completed with propofol (Propofol Lipuro 10 mg/mL; B. Braun, Germany) administered intravenously to effect, and anesthesia was maintained with isoflurane (Isoflutek 1,000 mg/g; Laboratorios Karizoo S.A., Barcelona, Spain) delivered in 70% oxygen. Continuous intraoperative monitoring included pulse oximetry, end-tidal CO_2_, rectal temperature, electrocardiography, and Doppler systolic arterial pressure.

**Fig. 1:**
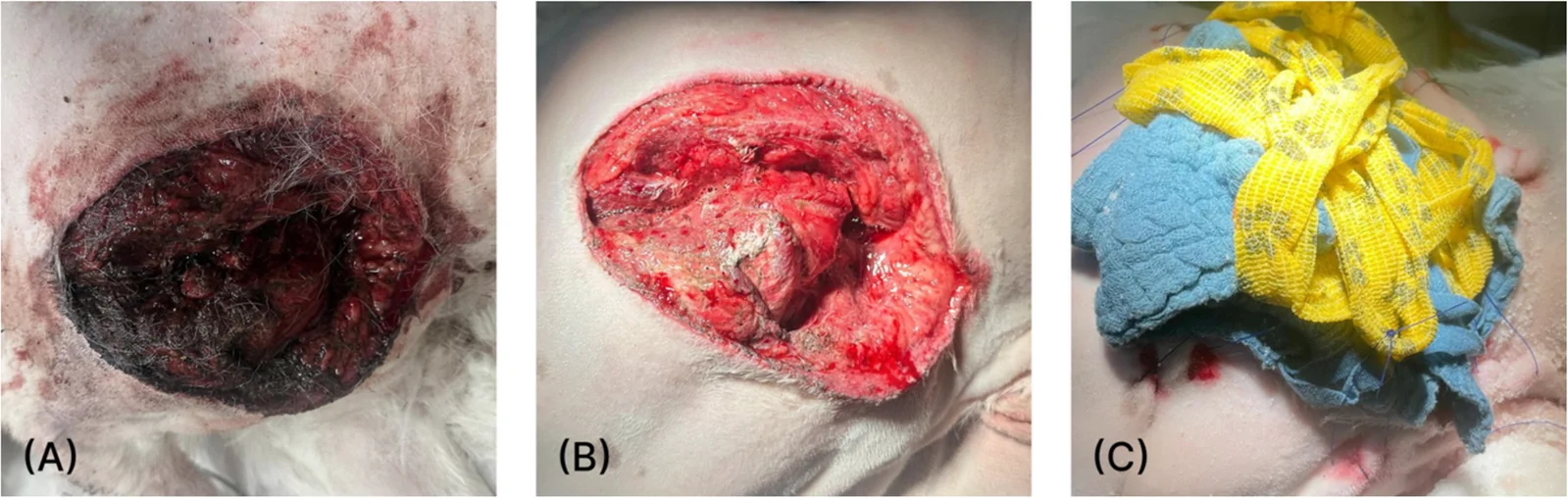
**A** Traumatic wound at presentation, showing extensive tissue devitalization and contamination. **B** Appearance of the wound following surgical debridement. **C** Placement of a tie-over bandage

Ischemic muscle and fascia were surgically debrided (Fig. 1A, B). Although the wound was less than 6 h old and classified as contaminated, lavage and mechanical removal did not eliminate all debris, making primary closure inappropriate. A small amount of sugar was applied, as it can help reduce bacterial load in contaminated, exudative wounds (Mphande et al. 2007). Its strong osmotic effect inhibits the growth of organisms such as *Escherichia coli*, *Pseudomonas aeruginosa*, and *Streptococcus*. In addition, sugar promotes mild superficial debridement, decreases malodor, and supports the early formation of granulation tissue (Chirife et al. 1983). These properties make it a simple, inexpensive, and accessible option for temporarily improving wound conditions. Due to the extensive defect on the proximal forelimb, an area where conventional bandages tend to slip, a tie-over bandage was applied for more stable coverage (Fig. 1C). This technique ensured secure wound protection and maintained an optimal environment for ongoing care. The dog recovered uneventfully from anesthesia and was transferred to the ICU for intensive monitoring and postoperative management. Antimicrobial and analgesic therapy continued throughout hospitalization. The dog was re-anesthetized for daily wound evaluation and tie-over bandage replacement during the first four days, followed by every three days for an additional week.

**Fig. 2:**
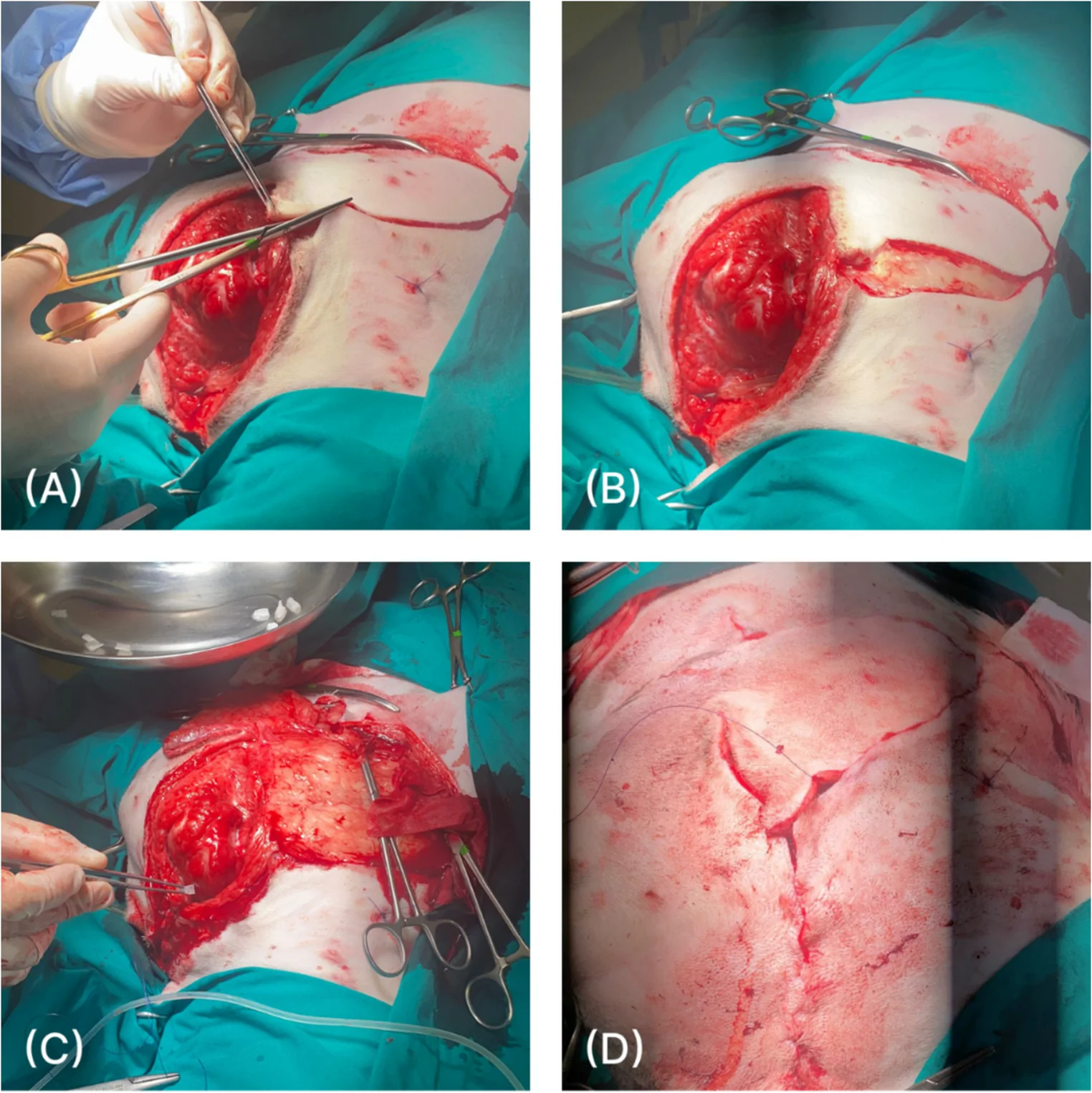
**A**,** B** Transpositional flap procedure. **C** Placement of BGAuSP-Alg-MCC composite within the defect. **D** Closure of the defect

Once the degree of wound contamination had markedly decreased and the local tissue conditions were considered satisfactory, definitive wound closure was achieved (Fig. 2). A microbiological sample subsequently tested positive for *Pseudomonas aeruginosa*, which was susceptible to amikacin but resistant to cefotaxime. Based on the antimicrobial susceptibility results, cefotaxime was discontinued, and amikacin was maintained as the sole antimicrobial therapy. Under general anesthesia, using the same protocol described previously, the dead space was filled with BGAuSP-Alg-MCC composite, followed by application of Hydrosorb^®^ Gel to maintain wound hydration and support early granulation. The defect was then covered with a transpositional skin flap (Fig. 2C, D). A Penrose drain was placed for 24 h to prevent fluid accumulation, with no significant drainage observed. Anesthesia and recovery were uneventful, and the dog remained clinically stable during the following day.

### Composite preparation

Firstly, the spherical gold nanoparticles (AuSP) was synthesized, followed by BGAuSP (60SiO_2_·31.85CaO·8P_2_O_5_·0.15Au_2_O mol%), using the Turkevich–Frens method (Frens 1973) and the sol–gel method, respectively, as described in previous studies (Magyari et al. 2017; Tóth et al. 2022). The gold amount introduced into the glass sample corresponded to 0.09 at% in the BGAuSP. The BGAuSP was introduced into Alg-MCC biopolymer composites using a synthesis described in a previous study (Feraru et al. 2025). The weight ratio of alginate-cellulose is 1:0.15, and the weight ratio of BGAuSP in the Alg-MCC composite is 12 wt%. For the synthesis details, structural and morphological properties, the Supplementary Materials can be consulted.

### Computed tomography protocol and assessment

Computed tomography (CT) examinations were performed immediately after definitive reconstruction and at 1 and 3 months postoperatively using a Siemens Somatom Scope system. The right forelimb and shoulder region were included in the field of view. The acquired images were stored in Digital Imaging and Communications in Medicine (DICOM) format on the Siemens workstation and archived on the PACS server. For this case report, CT assessment was qualitative and considered the position and conspicuity of the reconstructed/implant region, attenuation relative to adjacent muscle, surrounding soft-tissue changes, and evidence of complications. Standardized region-of-interest Hounsfield unit (HU) measurements and volumetric segmentation were not available for analysis; therefore, mean HU values and quantitative implant-volume changes are not reported.

### Histology and immunohistochemistry

At 3 months post-implantation, tissue samples were obtained using a punch biopsy technique. The specimens were fixed in 10% neutral-buffered formalin for 24 h and routinely processed using a Thermo Scientific Citadel 2000 tissue processor. Paraffin-embedded samples were sectioned at a thickness of 3 μm and stained with hematoxylin and eosin (H&E) and Masson’s Trichrome. For immunohistochemical evaluation, heat-induced epitope retrieval was performed at pH 9 using Novocastra™ Epitope Retrieval Solution (1:10 dilution) for 30 min. Immunolabeling was performed using the Dako EnVision Flex + Kit (Mouse, high pH) according to the manufacturer’s instructions. The primary antibodies used were α-smooth muscle actin (α-SMA; 1:100 dilution, polyclonal, ab5694, Abcam) and CD31 (ready-to-use, clone: JC70A, Leica Biosystems). Images were acquired using an Olympus SP-350 camera and Stream Basic imaging software, version 1.5.1 (Olympus Corporation, Tokyo, Japan).

### Postoperative course and follow-up

**Fig. 3:**
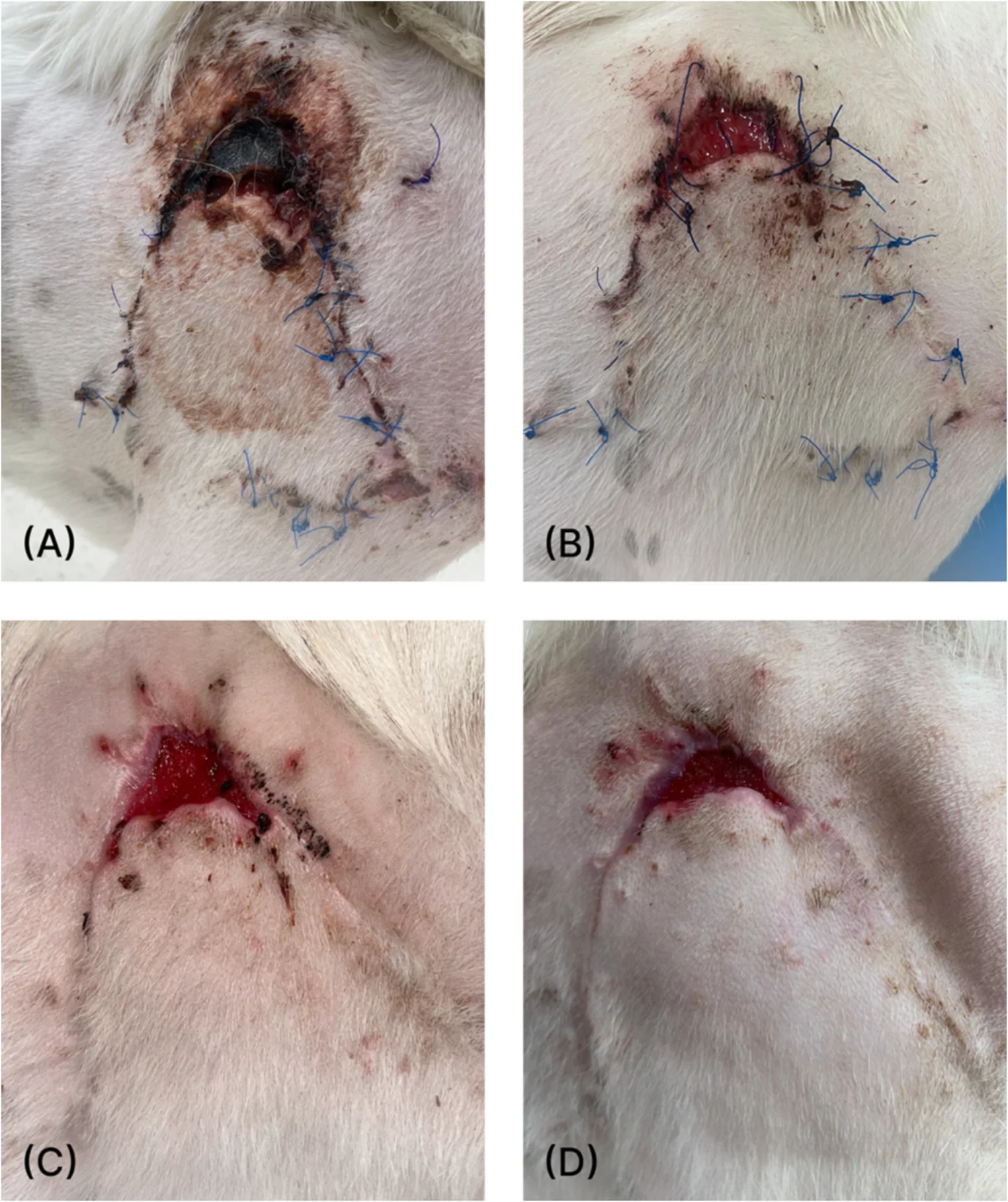
**A** Postoperative appearance of the wound 7 days after placement of the transposition flap and BGAuSP-Alg-MCC composite, showing partial skin necrosis and areas of incisional tension. **B** Progressive wound contraction and improved tissue viability observed 6 days later. **C** Continued healing with granulation tissue formation and reduction in wound size. **D** Further epithelialization and wound contraction, demonstrating steady progression toward wound closure

Seven days after placement of the transposition flap and composite, a small area of superficial necrosis was observed at the upper portion of the flap (Fig. 3A). A subsequent microbiological sample was culture-negative, making active bacterial contamination less likely. Clinically, the lesion was considered more consistent with local ischemia due to insufficient vascularization. The compromised region was surgically debrided and allowed to heal by secondary intention (Fig. 3B-D). No further infection, necrosis, or wound dehiscence was observed. Orthopedic assessment included visual gait observation and manual range-of-motion evaluation, with no obvious abnormalities detected.

**Fig. 4:**
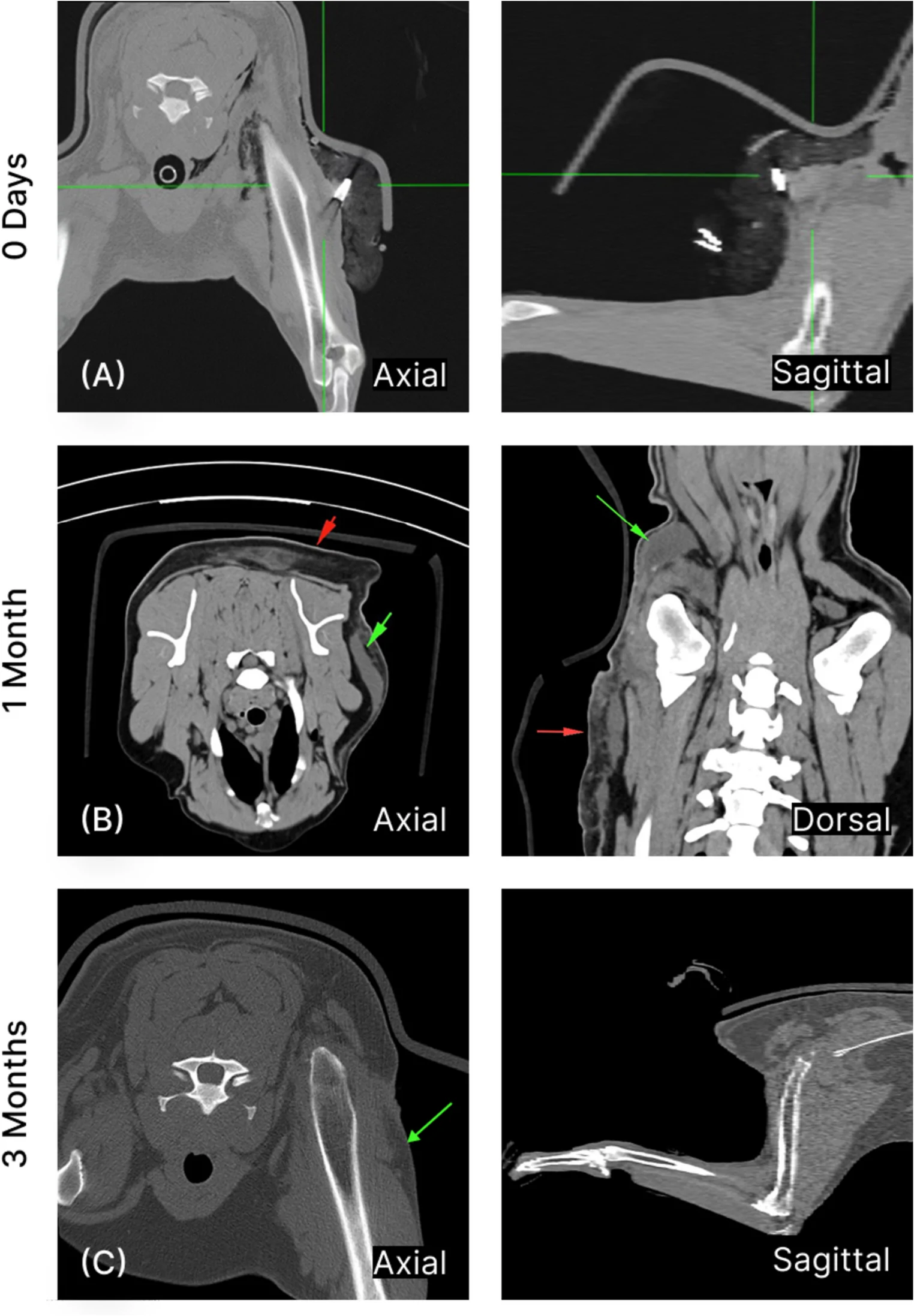
**A** Axial and sagittal CT images obtained immediately after definitive reconstruction, showing the focal hyperattenuating BGAuSP-Alg-MCC composite within the right shoulder defect. **B** Axial and dorsal CT images obtained 1 month postoperatively; green arrows indicate the reconstructed/implant region, whereas red arrows indicate the thin hyperattenuating band in the overlying subcutaneous tissue interpreted as mild localized panniculitis. **C** Axial and sagittal CT images obtained 3 months postoperatively; the green arrow indicates the repaired muscle region, whose attenuation approaches that of adjacent muscle

Immediately after definitive reconstruction, CT demonstrated the BGAuSP-Alg-MCC composite within the right shoulder muscular defect as a focal hyperattenuating region (Fig. 4A). On axial and sagittal reconstructions, the material was clearly delineated between the remaining muscle margins. No early fluid collection, abnormal gas pattern, or implant displacement was identified. At 1 month, the reconstructed/implant region remained mildly hyperattenuating relative to adjacent muscle (Fig. 4B; green arrows) but was less sharply delineated than immediately after surgery. This qualitative change was compatible with progressive incorporation or remodeling but was not sufficient to quantify material persistence or resorption. A thin, well-defined hyperattenuating band in the overlying subcutaneous tissue (Fig. 4B; red arrows) was interpreted as mild localized postoperative panniculitis without deeper extension.

At 3 months, the repaired muscle region (Fig. 4C; green arrow) appeared more homogeneous, and its attenuation approached that of adjacent muscle. The qualitative CT appearance was compatible with ongoing tissue remodeling. However, in the absence of standardized HU measurements and volumetric segmentation, CT could not determine the relative contributions of tissue integration, biomaterial persistence, and biomaterial resorption. No abscess, seroma, extensive surrounding soft-tissue inflammation, or imaging evidence of ongoing infection was identified at the 3-month CT examination.

**Fig. 5:**
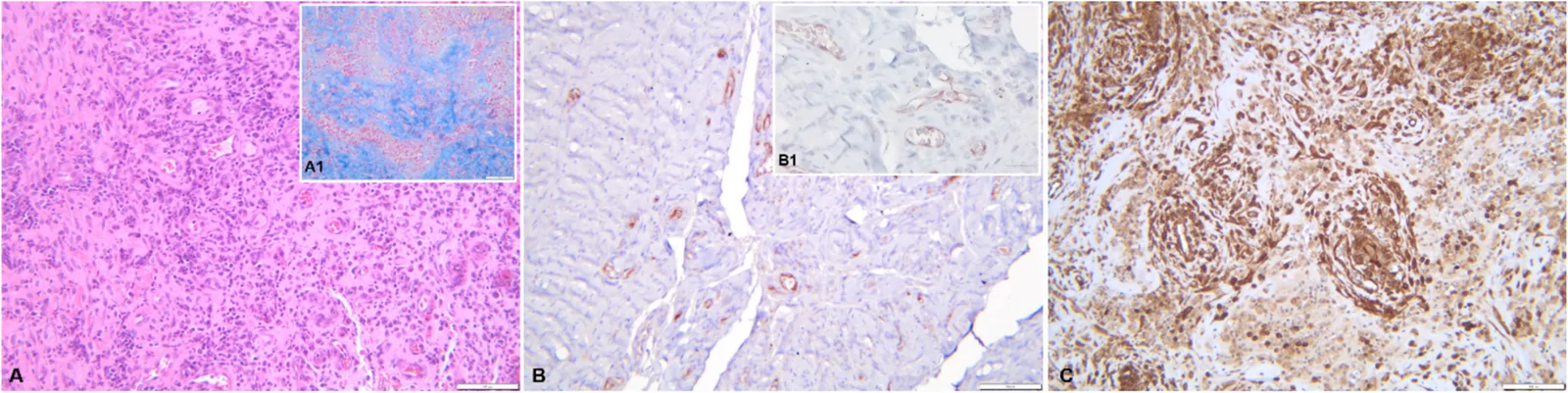
Microscopic features of the wound treated with BGAuSP-Alg-MCC. **A** Granulation tissue composed of collagen fibers, plump mesenchymal cells, and newly formed blood vessels is highlighted using Masson’s Trichrome stain (**A1**). This tissue is admixed with a moderate inflammatory infiltrate consisting predominantly of macrophages, with fewer neutrophils, lymphocytes, and plasma cells. H&E, scale bars: 100 μm (A and A1). **B**, **B1** Positive cytoplasmic immunoexpresion of CD31 in endothelial cells. Scale bar: 100 μm (**B**) and 20 μm (B1). **C** Strong cytoplasmic immunolabeling for α-SMA in pericytes, scale bar: 100 μm (**C**)

At 3 months post-implantation, histological examination revealed wound-associated granulation tissue with a moderate inflammatory infiltrate. The granulation tissue contained plump mesenchymal cells, moderate collagen deposition, and small, newly formed blood channels (Fig. 5A). The inflammatory cells consisted of moderate numbers of macrophages and neutrophils, along with scattered small lymphocytes and plasma cells. Immunohistochemical analysis confirmed angiogenesis, demonstrated by strong cytoplasmic CD31 expression in endothelial cells (Fig. 5B), which were surrounded by α-SMA-positive cells interpreted as pericytes (Fig. 5C). Analysis of CD31 and α-SMA-stained tissues revealed the presence of mature, well-formed blood vessels.

At discharge, oral amoxicillin-clavulanate (14 mg/kg PO q12h; Clavaseptin, Biotur) was prescribed for antimicrobial therapy, together with tramadol (4 mg/kg PO q8h; Tramvetol, Altius) and meloxicam (0.1 mg/kg PO q24h; Metacam, Boehringer Ingelheim) for 5 days for analgesia. The remaining superficial wound was managed at home with daily cleansing using sterile saline, and an Elizabethan collar was prescribed until complete healing. As the owner was unable to return to the clinic for weekly follow-up visits, the patient’s clinical progress was monitored through daily telephone contact until complete wound closure. The owner was asked about appetite, vocalization, mobility, response to palpation, demeanor, general activity, and wound status. No further wound dehiscence was reported.

## Discussion

Our previous study (Feraru et al. 2025) demonstrated the potential of BGAuSP-Alg-MCC to promote skeletal muscle regeneration, with regenerative effects observed both in vitro and in vivo. Based on these findings, this composite was selected for further investigation as a candidate biomaterial for VML treatment. The presence of CD31-positive endothelial cells and α-SMA-positive perivascular cells supports the formation of vascularized tissue with mural-cell investment, consistent with vascular development and ongoing tissue remodeling. Adequate vascularization is an important component of skeletal-muscle repair, as the presence of blood vessels ensures sufficient vascular support and helps maintain cell viability during muscle tissue growth and structural organization (Levenberg et al. 2005; Forbes and Rosenthal 2014). These findings therefore provide encouraging evidence of a vascularized reparative response within the reconstructed region. However, CD31 and α-SMA are not myogenesis-specific markers and do not distinguish newly regenerated muscle from tissue integration, granulation, or remodeling around the implanted material. Because myofiber-specific immunohistochemical markers and quantitative myofiber analysis were not performed, the present findings should be interpreted as evidence of vascularized tissue repair rather than definitive proof of the formation of functional new muscle fibers. While previous studies suggest that VML repair may be limited when scaffolds lack satellite cells or growth factors, the present findings suggest that the BGAuSP-Alg-MCC composite may support a vascularized reparative response and tissue remodeling in skeletal muscle, although its capacity to promote de novo muscle regeneration requires further investigation (Garg et al. 2014; Aurora et al. 2015).

Gold nanoparticles have attracted increasing interest in tissue repair because of their physicochemical and biological properties and their potential to act as delivery systems for therapeutic agents (Nukaly and Ansari 2023). Experimental studies in animal models have reported improved wound healing following gold nanoparticles-bioactive glass-based interventions compared with controls (Mârza et al. 2019, 2021). Proposed mechanisms include modulation of transforming growth factors, inflammatory cytokines, and angiogenesis-related pathways, together with antibacterial and antioxidant effects (Chen et al. 2023; Zhang et al. 2024). In addition, gold nanoparticles have been associated with enhanced collagen formation and organization, which may contribute to improved wound closure and reduced scar formation (Liu et al. 2012).

The staged clinical strategy combined serial debridement, sugar dressing, Hydrosorb^®^ gel, a transposition skin flap, and the BGAuSP-Alg-MCC composite. The favorable clinical course should therefore be interpreted as the result of this combined reconstructive approach, rather than as an effect of the biomaterial alone. Nevertheless, the incorporation of BGAuSP-Alg-MCC was associated with progressive wound healing and tissue remodeling within the reconstructed region. The moderate inflammatory infiltrate observed at 3 months also requires cautious interpretation. In the absence of a control group and quantitative inflammatory scoring, it may reflect ongoing wound remodeling, a host response to the implanted material, or a combination of both.

Limitations include the single-case design, absence of a control group, unknown long-term outcomes beyond 12 weeks, and possible subtle functional deficits undetected without advanced gait analysis. Variability in scaffold degradation, mechanical stability, and ion-release kinetics may also influence regenerative responses.

Future investigations should focus on larger, controlled cohorts to validate these findings and to better define the role of the BGAuSP-Alg-MCC composite in VML repair. Long-term follow-up and objective functional assessments are recommended to further characterize regenerative outcomes and durability. Additionally, optimization of scaffold composition and integration with supportive biological therapies may enhance muscle regeneration in extensive defects.

## Conclusion

In conclusion, this single case demonstrates the technical feasibility of using BGAuSP-Alg-MCC composite as an adjunct to transpositional flap reconstruction in a canine patient with complex volumetric muscle loss (see Supplementary Video). Over 12 weeks, the combined treatment was associated with progressive wound healing, a qualitative CT appearance compatible with remodeling of the reconstructed region, and vascularized granulation tissue at histology. Moderate inflammation remained present at 3 months. Because the patient received multiple concurrent interventions and no control treatment was available, the observed outcome reflects the combined reconstructive strategy, making the independent contribution of the BGAuSP-Alg-MCC composite difficult to determine. Nevertheless, the combined approach was associated with progressive wound healing, favorable clinical recovery, qualitative CT changes compatible with tissue remodeling, and vascularized granulation tissue at 3 months. These findings support the clinical feasibility of incorporating BGAuSP-Alg-MCC into flap-based reconstruction of severe muscular defects and provide a basis for further investigation of its potential role in tissue repair. Although the composite has been previously investigated in a controlled rat VML model, further studies in clinically relevant large-animal models with larger cohorts and longer follow-up are warranted to evaluate material persistence and degradation, host response, functional recovery, and the specific contribution of the composite to tissue regeneration.

## Supplementary Information

Below is the link to the electronic supplementary material.

Supplementary Material 1

Supplementary Material 2

## Data Availability

The original contributions presented in the study are included in the article; further inquiries can be directed to the corresponding author/s.
