# Supplementary material for: Management of volumetric muscle loss in a dog using bioactive glass–alginate–cellulose composite and a transpositional flap: a case report
Source: Vet Res Commun. 2026 Oct 9;50(6):624. doi: 10.1007/s11259-026-11557-x (PMC13652933; doi:10.1007/s11259-026-11557-x)
Supplement: Supplementary file 1 [file 11259_2026_11557_MOESM2_ESM.pdf]

Supplementary Materials

Management of volumetric muscle loss in a dog using bioactive glass–alginate–cellulose composite and a transpositional flap: a case report

Alina Zăvoi[1], Tom Ablassmaier[1], Bianca Pop[1], Ștefana Mureșan[1], Andrada Negoescu[1], Felix Lucaci[1], Robert Purdoiu[1], Alexandra Feraru[2,3] Klara Magyari[2,4,*], Lucia Bel[1,*], Mădălina Dragomir[1], Liviu Oana[1], Marian Taulescu[1], Alexandra Dreancă[1]

[1]Faculty of Veterinary Medicine, University of Agricultural Science and Veterinary Medicine, 400372 Cluj-Napoca, Romania

[2]Nanostructured Materials and Bio-Nano-Interfaces Center, Interdisciplinary Research Institute on Bio-Nano-Sciences, Babeș-Bolyai University, 400271 Cluj-Napoca, Romania

[3]Doctoral School in Physics, Faculty of Physics, Babeș-Bolyai University, M. Kogălniceanu 1, 400084 Cluj-Napoca, Romania

[4]INSPIRE Research Platform InfoBioNano4Health & Biomedical Imaging, Babeș-Bolyai University, 400084 Cluj-Napoca, Romania

Materials

Hydrogen tetrachloroaurate (III) hydrate ($HAuCl_4 \cdot 3H_2O$, 99.99%, Sigma-Aldrich), trisodium citrate dihydrate (ACS, 99.0%), and Pluronic F127 (powder, BioReagent, suitable for cell, Sigma-Aldrich) were used for gold nanoparticles (AuSP) synthesis.

The precursors used for the bioactive glass (BG) synthesis were tetraethyl orthosilicate (TEOS, ≥99%, Merck), triethyl phosphate (TEP, ≥99%, Merck), and calcium nitrate tetrahydrate ($Ca(NO_3)_2 \cdot 4H_2O$, ≥99%, Lach-Ner), hydrolyzed in the presence of nitric acid (HNO_3, 65%).

For the synthesis of composites, sodium alginate (Na-Alg) and cellulose microcrystal (MCC) polymeric powder were obtained from Sigma-Aldrich. Cross-linking was performed with calcium carbonate ($CaCO_3$, >99%, Nordic Chemicals) and D-(+)-gluconic acid δ-lactone (GDL, ≥99%, Sigma-Aldrich).

Ultrapure water and absolute ethanol were used throughout the whole experimental process. All chemicals were used as received without further purification.

Spherical gold nanoparticles synthesis

The spherical gold nanoparticles (AuSP) were obtained using the Turkevich-Frens method (Frens, 1973). $HAuCl_4 \cdot 3H_2O$ solution was prepared (10^{-3} M). After this, the solution was heated to the boiling point of the water. Afterward, 38.8×10^{-3} M trisodium citrate solution was instantly added to the boiling gold precursor solution using a 10:1 volume ratio. The whole procedure continued for 30 min, and then it was cooled to room temperature. For further application, AuSP were stabilized using a Pluronic F127 block copolymer solution of 0.5×10^{-3} M. The solution containing the AuSP was stirred for 20 min, followed by an aging process of 24 hours to ensure the adherence of the polymer molecules. The excess of Pluronic F127 was removed by collecting the supernatant liquid from the centrifugation tubes, which were held for 30 min at 12000 rpm. The authors would like to mention also that all the stabilized AuSP were used during the preparation process of the bioactive network. Consequently, the real Au content can be considered the initial use done.

Synthesis of bioactive glass with spherical gold nanoparticles

The gold nanoparticles-bioactive glass (BGAuSP, $60SiO_2 \cdot 31.85CaO \cdot 8P_2O_5 \cdot 0.15Au_2O$ mol%) was synthesized *via* a sol-gel method, as described in our previous study (Magyari et al., 2015, 2017). The gold amount is conventionally indicated in the oxidic form Au_2O; the gold content amount is 0.09at%. The reactants were added consecutively after 1-hour intervals under continuous stirring. Gelation took place in 24 hours at 37°C, then the as-prepared gel was aged for 3 days at 37°C. The aged gel was dried at 110°C for 24 hours and then stabilized by applying a heat treatment at 500°C for 2 hours. Before characterization, the samples were ground using a ceramic mortar.

Synthesis of bioactive glass-alginate-cellulose composite

The composite synthesis was based on the ionic cross-linking method. The 1.5 % (w/v) Na-Alg and MCC polymeric solutions were dissolved in ultrapure water (25 mL) under continuous stirring. To the alginate-cellulose solution, 5 mL of calcium carbonate ($CaCO_3$) with a concentration of 112.5 mM was added. The mixture was continuously stirred until homogenization (30 min). The BGAuSP were ultrasonicated for 1 hour to obtain a uniform particle size, then added to the polymeric solution. To initiate the ionization of $CaCO_3$, 5 mL GDL solution was added to the mixture, and the solution was immediately poured into the well plate. The weight ratio of the Alg-MCC composite was 1:0.15. The BGAuSP were added to the polymeric mixture in a weight ratio of 12 wt% and abbreviated as BGAuSP-Alg-MCC.

Methods

The Scanning Electron Microscopy (SEM) micrographs were recorded with a Hitachi S-4700 Type II cold field emission scanning microscope (Tokyo, Japan) operated at an acceleration voltage of 10 kV.

The X-ray diffraction pattern was obtained using a Shimadzu X-ray diffractometer (XRD 6000, Kyoto, Japan), operated with CuKα radiation (λ=1.54 Å) and a Ni filter. The diffraction patterns were recorded in the 2θ range 10°-80° with a scan speed of 2°/min.

Fourier Transform Infrared (FT-IR) absorption spectra were recorded in reflection configuration with a Jasco 6200 FT-IR (Jasco, Tokyo, Japan) spectrometer using the well-known KBr pellet technique, and the following parameters: room temperature, 400–4000 cm^{-1} range, 4 cm^{-1} spectral resolution.

Supplementary figures

The pore size of the BGAuSP-Alg-MCC composites was 97±7µm (Fig. S1B).

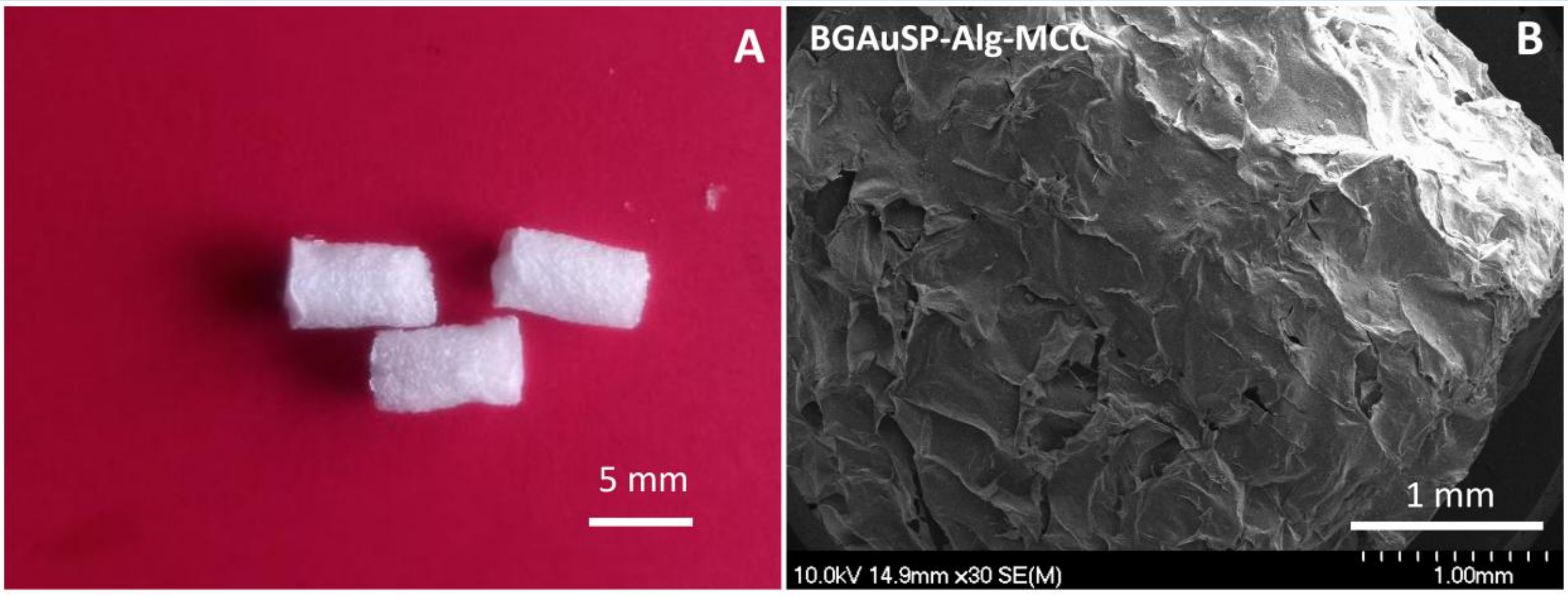

Fig. S1 Digital images (**A**) and SEM micrograph (**B**) of the BGAuSP-Alg-MCC composite

The XRD pattern of BGAuSP revealed the amorphous structure, whereas the presence of gold nanocrystals in the glass is proven by the reflection at 2θ=38.1° (Fig. S2A). The FT-IR spectra of the bioactive glasses have characteristic absorption bands of the silicate network, and the spectral characteristics are not influenced by the AuSP (Fig. S2B). In the BGAuSP-Alg-MCC composite spectra, the appearance of the Si–O–C vibration at 877 cm^{-1} indicates the bond between biopolymers and BGAuSP. The XRD pattern and FT-IR spectra of the alginate-cellulose (Alg-MCC) composite were introduced for comparison purposes.

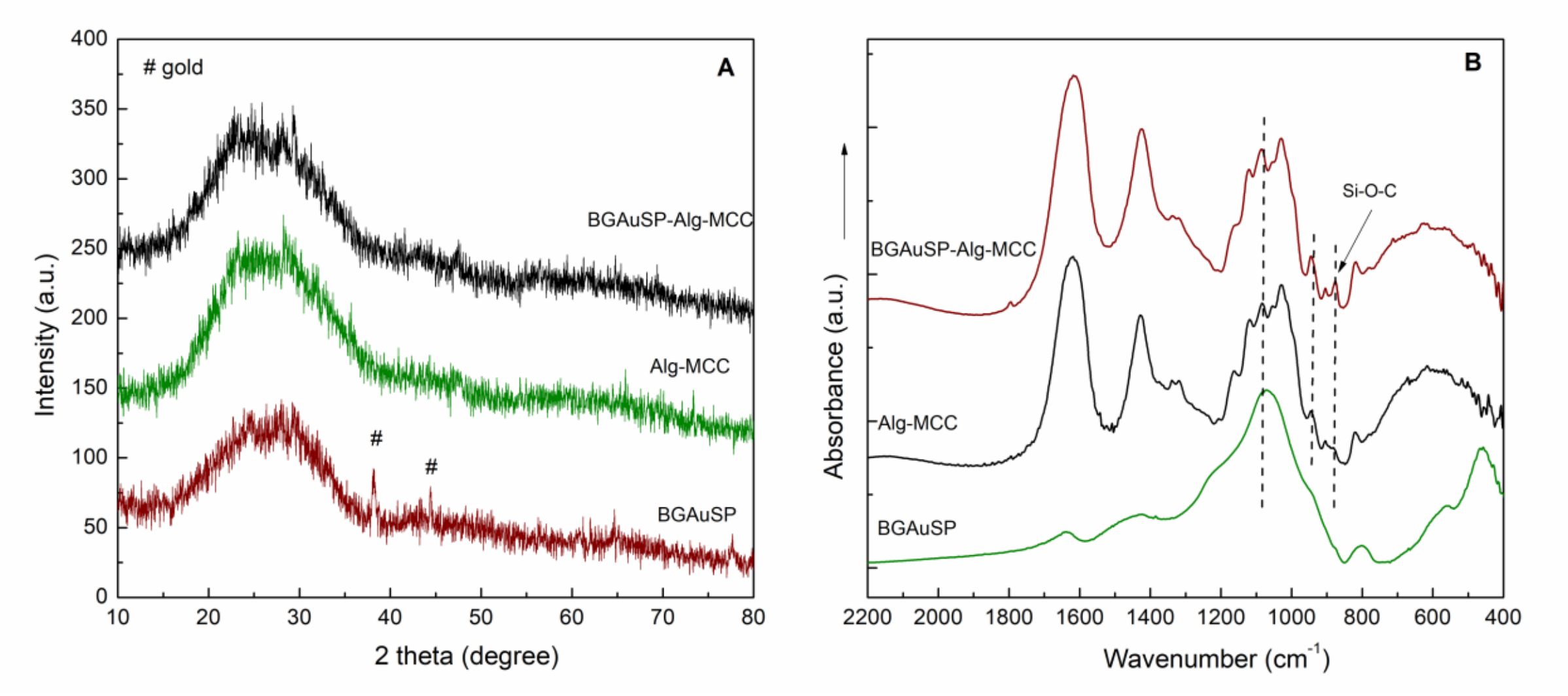

Fig. S2 XRD pattern (A) and FT-IR spectra of the BGAuSP, Alg-MCC, and BGAuSP-Alg-MCC composites

Frens G (1973) Controlled Nucleation for the Regulation of the Particle Size in Monodisperse Gold Suspensions. Nature Physical Science volume 241:20–22

Magyari K, Baia L, Vulpoi A, et al (2015) Bioactivity evolution of the surface functionalized bioactive glasses. J Biomed Mater Res B Appl Biomater 103:261–272. https://doi.org/10.1002/jbm.b.33203

Magyari K, Nagy-Simon T, Vulpoi A, et al (2017) Novel bioactive glass-AuNP composites for biomedical applications. Materials Science and Engineering C 76:. https://doi.org/10.1016/j.msec.2017.03.138
